# Confirmation of the Prognostic Value of Foxp3+ Cells in Canine Mammary Tumors

**DOI:** 10.3390/ani13030505

**Published:** 2023-01-31

**Authors:** Francesca Parisi, Francesca Millanta, Marika Nicastro, Iacopo Vannozzi, Alessandro Poli

**Affiliations:** Department of Veterinary Sciences, University of Pisa, Viale Delle Piagge n. 2, 56124 Pisa, Italy

**Keywords:** canine mammary tumors, FoxP3, immunohistochemistry, prognostic markers, TME

## Abstract

**Simple Summary:**

Foxp3+ cells have immunosuppressive properties that can interfere with beneficial anti-tumor immunity, enabling tumors to elude the host antitumor immune response. It has already been suggested that these cells play a role in canine mammary tumor progression, but the literature on this topic is poor. Our work aims to investigate Foxp3+ cells in 59 canine mammary tumors by immunohistochemistry and to evaluate associations with clinicopathological, immunohistochemical features, and overall survival (OS). Our findings confirm that the number of Tregs is significantly higher in canine mammary carcinomas than adenomas and that a high number of Foxp3+ cells were associated with negative prognostic factors and shorter overall survival (OS).

**Abstract:**

Foxp3+ cell counts were evaluated by immunohistochemistry in 59 canine mammary tumors, 20 adenomas, and 39 carcinomas in three different compartments: intratumoral, within the adjacent stroma, and in the distant stroma. Foxp3+ lymphocyte counts were compared with histotype, grading, presence of lymphatic invasion, immunohistochemical expression of estrogen and progesterone receptors, expression of c-erbB-2, and the overall survival (OS). Our findings confirmed that Foxp3+ cells were significantly higher in canine mammary carcinomas compared to adenomas. A significantly higher number of Foxp3+ cells were detected in grade III carcinomas compared to grade II carcinomas, as well as in tumors with lymphatic invasion and loss of ER-expression. Finally, a high number of Foxp3+ cells was associated with poor prognosis. In conclusion, our findings highlighted the association of Foxp3+ lymphocytes with negative clinicopathological features and shorter overall survival (OS), thus confirming the role of Tregs as a negative prognostic marker in canine mammary carcinomas.

## 1. Introduction

Regulatory T (T reg) cells are a subset of T lymphocytes involved in the modulation of immune response and in the maintenance of immune homeostasis. They are components of the so-called Tumor Microenvironment (TME), which is defined as the complex and dynamic environment in which tumor exist, characterized by cellular and acellular components involved in cancer progression [1,2]. Endothelial cells, immune cells (granulocytes, lymphocytes, and macrophages), and fibroblasts, together with acellular components (such as glycoproteins, collagens, and enzymes) influence cancer development [2]. Similarly, tumoral cells influence TME via paracrine signals [3]. Due to its strong influence in tumor progression, TME has become a pivotal point of research in oncology in order to try to manage tumor cells by targeting the involved components. It has been largely reported that Tregs usually act as suppressors of antitumor immune response, promoting neoplastic progression [4,5]. In most human solid tumors, including breast cancer [6,7,8], Tregs have been associated with a worse prognosis, and the perception of these cells as a negative marker is prevalent [5]. Nevertheless, Treg lymphocytes were also subjected to the action of other environmental factors and paracrine signals that may affect their function. For this reason, they have been associated with improved survival times [5]. This is true for some type of tumors, especially those showing prominent chronic inflammation, but also for some tumors, such as breast cancer [9], in which Treg lymphocytes were previously associated with a poor prognosis, underlining the complexity of Treg as a biomarker. Veterinary studies on mammary carcinomas are few and reported a putative correlation of Tregs cells with aggressiveness [10] and poor prognostic factors [11,12]. However, in view of the complexity of Treg as a biomarker which was highlighted by the contrasting reports in human literature, further studies are needed to confirm or reject this hypothesis. In this view, the aim of this study was to assess the presence of Treg cells in canine mammary carcinoma through the identification of Foxp3, their currently most reliable marker [13], and the association of Treg cells with various clinicopathological and immunohistochemical features and overall survival (OS).

## 2. Materials and Methods

### 2.1. Animals and Tissue Samples

Fifty-nine samples from surgically excised mammary gland tumors were selected from the Tumor Registry of the Department of Veterinary Science of the University of Pisa and included in the study. Tumors belonged to female dogs (mean age = 9 years, range = 4–14 years) in cases for which surgery was the only therapeutic option. For each subject, medical history and anamnesis, including breed, age, and gender, were collected. When multiple malignant neoplasms were diagnosed, the tumor with the most aggressive clinico-pathological features was selected for the study [14]. Normal mammary gland tissue specimens were collected during routine necropsy from five bitches that had died to causes unrelated to mammary tumors after the owner’s consent. In order to record the presence of distant organ metastases and the recurrence of primary tumors, a 2-year post-surgery follow-up study (730 days) was carried out by performing clinical examinations and tumor staging at 6, 12, 18, and 24 months after surgery. Dogs that had died within this follow-up period were subjected to necropsy examination to confirm tumor-related death. For the subjects who survived, the OS was calculated as the number of days from the surgery to the latest examination. For those who died within these 730 days due to complications related to mammary neoplasms, the OS was expressed by the number of days from surgery to the death of the animals.

### 2.2. Histopathological Investigation

Tissue samples were fixed in 10% neutral buffered formalin and then embedded in paraffin wax. Four-micrometer tissue sections from mammary tumors stained with hematoxylin and eosin (H&E) were investigated by two experienced pathologists under blinded conditions for the anamnesis for histopathological investigations. The classification of the tumors was carried according to Goldschmidt et al. [15]. Tumors displaying multiple features were classified according to the most malignant histologic differentiation. Tumor grading was assessed according to Peña et al. [16]. Furthermore, lymphatic invasion was recorded.

### 2.3. Immunohistochemistry

The number and the localization of cells bearing Foxp3 antigen and the presence of estrogen receptors (ER), progesterone receptors (PR), and human epidermal growth factor receptors 2 (c-erbB-2) was investigated by immunohistochemistry.

Four-micrometer thick sections were de-waxed in xylene and rehydrated in a graded series of ethanol and water. Antigen retrieval was performed by placing the slide in a bath of citrate buffer of pH 6.0 in a microwave oven for 5 min at 750 W and 13 min at 350 W. The sections were cooled at room temperature and rinsed in TRIS Buffered Saline solution (TBS) at pH 7.6. Endogenous peroxidase activity was blocked via incubation with Peroxidase blocking solution^®^ (Dako, Glostrup, Denmark) for 10 min at room temperature. Non-specific binding was prevented by incubating each section with two drops of Ultra V-block^®^ (ThermoFisher Scientific; Waltham, MA, USA) for 5 min. The primary antibodies used in this study were an anti-estrogen receptor (ER, clone B-10, dilution 1:300, Abcam, Cambridge, UK), an anti-progesterone receptor (PR, clone PR4-12, dilution 1:100, Oncogene TM, Boston, MA, USA), a polyclonal anti-human epidermal growth factor receptor 2 (c-erbB-2, dilution 1:250, Dako, Glostrup, Denmark), an anti-mouse/human Foxp3 (clone eBio7979, dilution 1:100, eBioscience, San Diego, CA, USA). The primary antibodies were incubated overnight at 4 °C. The following day, after two washes, samples were incubated with a universal polyvalent biotinylated antibody (Horse anti-mouse/rabbit IgG RTU, Vector Laboratories, Burlingame, CA, USA) for 15 min. Antibody binding was detected using a streptavidin–peroxidase complex (Vector Laboratories, Burlingame, CA, USA) for 15 min. To develop the reaction, 3,3′-diamonibenzidine tetrahydrochloride was used (ImmPACT DAB Peroxidase Substrate Kit^®^, Vector Labs inc., Burlingame, CA, USA) and blocked with deionized water. Sections were counterstained with Mayer’s hematoxylin, dried, and covered with cover slips. Negative controls were performed by replacing the primary antibody with irrelevant, isotype-matched antibodies and an antiserum. Tissue samples from normal canine uterus (for anti-ER and anti-PR), canine mammary carcinoma known to react with c-erbB-2 antibody [16], and canine normal lymph nodes (for anti-Foxp3) were used as the internal positive control.

### 2.4. Quantification of Immunolabelling

Foxp3 IHC positive staining was recorded when a brown nuclear pattern of immunoreactivity was detected in lymphocytes. The number of Foxp3+ lymphocytes was analyzed in 10 high-power fields (2.4 mm^2^) with automated image-analysis software (LAS 4.10, Leica, Heerbrugg, Switzerland) in three different compartments: the intratumoral compartment, in the stroma adjacent to the tumor (distance between positive cell and tumor nest less than one tumor cell size), and in the stroma far from tumors (distance between positive cell and tumor nest more than one tumor cell size), as previously published [17]. The value of Foxp3+ cells was then expressed as mean value ± standard deviation (SD). Tumors were considered positive to ER (Figure S1) and PR (Figure S2) when more than 5% of tumoral cells showed nuclear staining [18]. Tumors with a complete membranous immunoreactivity to anti-c-erbB-2 antibody (Figure S3) in more than 10% were considered positive (overexpressing) [19].

### 2.5. Statistical Analysis

Statistical analysis was performed using the statistical package SPSS Advanced Statistic 21 (SPSS Inc, Chicago, IL, USA). The associations between Foxp3+ cells and the site of infiltrations; the histological subtype; the histological grade; the presence of lymphatic invasion of tumor cells; the expression of ER, PR, and c-erBb-2 receptors; the number of alive or dead subjects were determined using the Bonferroni corrected post-hoc ANOVA test. Due to the low number of samples within the groups of solid carcinomas and anaplastic carcinomas, these two groups were merged for statistical purposes. Statistical significance was based on a 5% (0.05) significance level. Cancer-specific overall survival analysis was performed using the Kaplan–Meier method and both the Tarone–Ware and the log-rank (Mantel–Cox) tests were used to evaluate the relationship between OS and other variables.

## 3. Results

### 3.1. Clinicopathological Data

Of the bitches included in the study, 28 were mixed breed, whereas the remaining 31 were pure breeds, among which Boxer (n = 5), German Shepherd (n = 3), English Setter (n = 3), Yorkshire Terrier (n = 3), Dobermann (n = 2), Labrador Retriever (n = 2), Cocker Spaniel (n = 3), Poodle (n = 3), Maremmano-Abruzzese Sheepdog (n = 1), Jack Russel (n = 1), Italian Volpino (n = 1), French Bulldog (n = 1), West Highland White Terrier (n = 1), Border Collie (n = 1), and Hound (n = 1) were recorded. The diagnosed histologic types were 20 (33.9%) canine mammary adenomas (CMAs) and 39 (66.1%) canine mammary carcinomas (CMCs), among which 14 (35.9%) were complex, 5 (12.8%) were simple tubular, 8 (20.5%) were tubulopapillary, and 12 (30.7%) were solid carcinomas (Table 1). According to the grading system, 30 (76.9%) CMCs were of grade I, 6/15.4%) CMCs were of grade II, and 3 (7,7%) were of grade III. Thirty CMCs (77%) did not show lymphatic invasion, whereas in nine CMCs (23%), lymphatic invasion in vessels around tumors was detected. Of the 39 subjects bearing mammary carcinomas, 9 (23%) died for the progression of the neoplastic disease before the end of the follow-up period, whereas 30 (77%) were still alive.

### 3.2. Expression and Number of Foxp3

**Figure 1:**
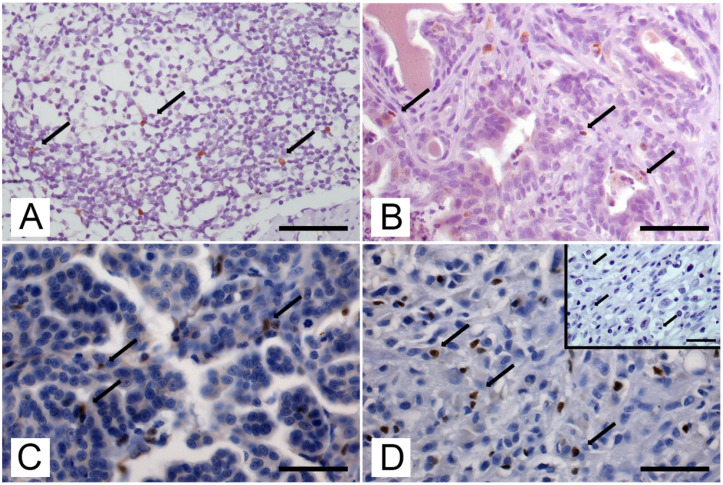
Immunohistochemical localization of Foxp3+ cells on lymph node tissue (positive control) and canine mammary tumors; labelled streptavidin biotin (LSAB) method IHC using an antibody anti-Foxp3 (eBioscience, clone eBio7979). The arrows point to exemplificative Foxp3+ lymphocytes. (**A**) Lymph node: positive lymphocytes in the mantle area of a lymphatic follicle. IHC: hematoxylin counterstain, scale bar = 100 μm. (**B**) Canine mammary adenoma: scattered Foxp3+ lymphocytes (arrows) infiltrating the tumor. IHC: hematoxylin counterstain, scale bar = 50 μm. (**C**) Canine mammary carcinoma: several Foxp3+ lymphocytes (arrows) infiltrating a tubular mammary carcinoma. IHC: hematoxylin counterstain, scale bar = 50 μm. (**D**) Canine mammary carcinoma: several Foxp3+ lymphocytes (arrows) infiltrating a solid mammary carcinoma. Inset: negative control. The arrows point to exemplificative Foxp3- lymphocytes. IHC: hematoxylin counterstain, scale bar = 50 μm.

Immunoreactivity to the anti-Foxp3 antibody was nuclear and was detected in lymph nodes (Figure 1A) and neoplastic mammary tissue (Figure 1B–D), whereas normal mammary gland samples were always negative. Foxp3+ cells were detected in 7/20 (35%) CMAs and in 37/39 (95%) CMCs, in the intratumoral compartment and in both adjacent and distant stroma. The mean number (±SD) of total Foxp3+ cells per HPF (high power fields) was 3.9 ± 6.2 in CMAs and 28.1 ± 33.8 in carcinomas CMCs. (*p* < 0.0001) In CMAs, for each compartment, the number of Foxp3 was 4.8 ± 7.3 in intratumoral localization, 3.1 ± 5.1 in adjacent stroma, and 3.8 ± 6.2 in distant stroma. Similarly, in CMCs, the number of Foxp3 was 67.6 ± 27.9 in intratumoral localization (*p* < 0.0001), 12.0 ± 15.9 in adjacent stroma (*p* = 0.18), and 4.7 ± 6.5 (NS) in distant stroma.

### 3.3. Associations of Foxp3 with Clinicopathological and Immunohistochemical Features and OS

The association between the number of Foxp3+ lymphocytes in the three compartments and the histopathological and immunohistochemical features was examined and is summarized in Table 1. The mean number of Foxp3 was higher in carcinomas than in adenomas in all compartments (*p* < 0.000 for the intratumoral compartment and *p* = 0.018 for the adjacent stroma). The number of Foxp3+ cells in the intratumoral compartment of CMCs ranged from a minimum of 0 to a maximum of 140, with a mean value of 67.2 ± 27.8. The mean value of Foxp3+ cells of the intratumoral, adjacent, and distant stroma was significantly higher in simple, solid than in complex carcinomas (*p* < 0.000) as well as than in simple tubular carcinomas (*p* = 0.01 for the intratumoral compartment, *p* = 0.000 for both adjacent and distant stroma). The mean number of Foxp3+ cells was significantly higher in grade III carcinoma than in grade I in all compartments (*p* < 0.000 in the intratumoral compartment and adjacent stroma, and *p* = 0.01 in distant stroma), whereas it was higher in grade III when compared to grade II carcinoma in the intratumoral compartment (*p* = 0.02) and adjacent stroma. In distant stroma, the number of Foxp3+ cells was slightly higher in grade II carcinoma, but this result was not statistically significant. Foxp3+ cell count in the intratumoral compartment was also higher in subjects showing lymphatic invasion (*p* < 0.000). Furthermore, the mean value of Foxp3+ lymphocytes was related to immunohistochemical features of mammary carcinoma, particularly the expression of hormones and C-erbB-2 receptors. The mean number of Foxp3+ cells was higher in tumor that did not show ER expression (*p* < 0.000 in the intratumoral compartment; *p* = 0.007 in the adjacent area; and *p* < 0.000 in the distant area). The same trend, even if not significative, was evident for samples that were PR-. Foxp3+ cells count was not statistically associated to c-erbB-2 expression.

**Figure 2:**
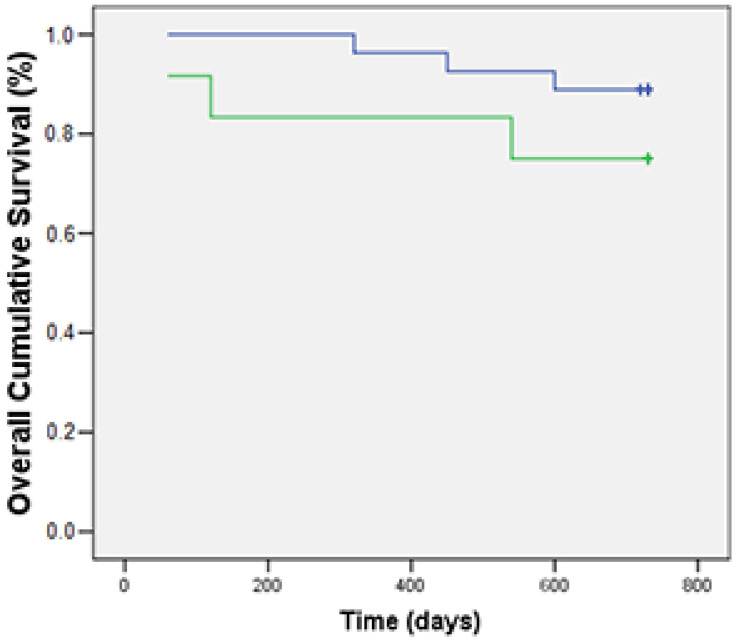
Kaplan–Meier estimates of OS in the examined canine mammary carcinomas (n = 39) according to the median number of FoxP3+ cells (67.2 ± 27.8). Subjects with a higher number of Foxp3+ cells (green line) show a shorter OS than those with higher number of Foxp3+ cells. Survival function *p* = 0.007.

Finally, the mean number of Foxp3+ cells was higher in tumors from dead subjects than in those from live ones (*p* = 0.017 in the intratumoral compartment). An increased number of Foxp3 was thus associated with shorter OS (Figure 2).

## 4. Discussion

TME has become a pivotal point in oncology due to the increasing evidence that the interaction between its components and tumor cells can affect tumor development and progression [20,21]. Currently, the evaluation of TME is among the tumoral features with the greatest prognostic value because it allows for the development of therapies and interventions to manage and treat the tumor [22,23]. Mammary tumors are the most common tumors in female dogs [24,25], but this group of neoplasms is so heterogeneous that prognosis cannot be easily assessed. In the last ten years, an increasing interest in the study of immune cells that make up the TME of the tumors has been highlighted [26,27,28,29,30], thanks also to personalized medicine and immune therapies that are gaining traction in human medicine [31]. Particularly, the role of lymphocytes is in the crosshair of the recent studies [28,32,33,34]. Among T lymphocytes, Tregs include CD4+, CD25+, and FoxP3+ cells physiologically involved in preventing harmful autoimmune response, but in this role, they can also interfere with antitumoral immune response [35,36,37,38]. Different authors suggested that Tregs are implicated in enhancing tumor progression [10,37,38,39,40]. Regarding mammary cancer, human studies on the role of FoxP3 have highlighted contrasting results, and the veterinary literature is poor. This study focused on the role of Foxp3+ cells in canine mammary carcinoma in three areas: the intratumoral compartment, the adjacent stroma, and the distant stroma. Our results showed that infiltration of Foxp3+ cells was low in normal mammary tissue, as already highlighted by previous authors [10,11], whereas only a small percentage of adenoma (35%) contained Treg lymphocytes. Conversely, almost all CMCs (98%) were characterized by Foxp3+ cell infiltrates, and the number of these cells was significantly higher in CMC than in CMA in all the three compartments. Among carcinomas, a high number of Foxp3+ cells was significantly correlated with more aggressive histotype and grading and with the presence of lymphatic invasion, which is in agreement with previous studies [10,11,12,41].

As already stated, mammary tumors are a complex and heterogeneous group of neoplasms that are characterized by multiple subtypes differing morphologically and molecularly, with consequently different biological behaviors and prognoses [42]. To better investigate breast cancer, some cancer-related molecular markers have been introduced in human medicine, among which ER, PR, and c-erbB-2 are the most significative [43]. Because CMC has been confirmed to be a good animal model for human breast cancer [44,45,46], the same human tumor-related markers were introduced to the studies of canine mammary neoplasms, and great efforts have been made and are still in progress to make these markers of common use in veterinary diagnostics [47]. Considering that, for a deeper overall view, the expression of ER, PR, and c-erbB-2 was also investigated, and the presence of T regs correlated to these markers. Foxp3+ lymphocytes were significantly higher in ER- tumors in all the three examined compartments, as already observed (even if without statistical significance) by Kim et al. [10]. Unlike PR expression, the evaluation of which acquires prognostic relevance only when associated with ER evaluation [48], ER expression is considered an independent prognostic marker [48]. For this reason, we can conclude that the association of a higher number of Foxp3+ cells with the lack of ER expression is a further clue of the negative prognostic value of T regs in canine mammary carcinoma. In our study population, a high number of Foxp3+ cells were also observed in PR- tumors, even without statistical significance.

The number of Foxp3+ cells did not differ among samples with different c-erbB2 receptor expression. Nevertheless, despite the fact that in human medicine, c-erbB-2 overexpression is routinely used as a prognostic and predictive factor [49] associated with aggressive clinicopathological features, significant reduced survival [50] recurrence, and metastasis [50,51] in BC patients, its role in veterinary medicine is still not clear. Studies on the prognostic value of c-erbB-2 in CMCs have been characterized by great variability with sometimes conflicting results, from which no agreement on the prognostic value of c-erbB-2 in CMCs emerges [47].

Statistical analysis on overall survival data highlighted an association between an increasing number of Foxp3+ cells and a worse prognosis, in agreement with what was observed by Oh et al. [41] and Carvalho et al. [11], thus confirming the role of Tregs as negative prognostic markers.

## 5. Conclusions

Our study supports the hypothesis that a high number of Treg cells may interfere with beneficial anti-tumor immune responses in canine mammary carcinomas. Our results thus suggest an association with negative tumoral prognostic factors, tumor aggressiveness, and malignant progression. The relationship between a high number of Foxp3+ lymphocytes and a shorter overall survival highlights the fact that the amount of Foxp3+ cells should be considered to be an important negative prognostic marker in these neoplasms.

## Figures and Tables

**Table 1:** Correlation between the mean of Foxp3+ cells (± SD) and clinicopathological and immunohistochemical features of canine mammary tumors.

	Foxp3 intra	*p*	Foxp3 adj	*p*	Foxp3 dist	*p*
	Mean ± SD		Mean ± SD		Mean ± SD	
Diagnosis				*p* < 0.05		NS
Adenoma (n = 7)	4.8 ± 7.3	*p* < 0.0005	3.1 ± 5.1		6.6 ± 0.97	
Carcinoma (n = 39)	67.2 ± 27.8		12.0 ± 15.9		10.98 ± 1.23	
Histotype CMCs						
CC (n = 14)	50.6 ± 17.3 ^a^	(a) *p* < 0.0005 (b) *p* > 0.05	5.0 ± 17.3 ^c^	(c) *p* < 0.0005	1.3 ± 2.4 ^d^	(d),(e) *p* < 0.0005
STC (n = 5)	46.2 ± 29.5 ^b^		0.0		0.0 ^e^	
STPC (n = 8)	69.6 ± 6.4		7.5 ± 5.6		2.7 ± 3.3	
SSC + AC (n = 12)	95.0 ± 24.0		28.8 ± 20.0		12 ± 6.9	
Grading						
Grade I (n = 30)	58.6 ± 21.5 ^f^	(f) *p* < 0.0005 (g) *p* < 0.05	7.5 ± 8.5 ^h^	(h),(i) *p* < 0.0005	2.8 ± 5.0 ^l^	(l) *p* < 0.05
Grade II (n = 6)	80.5 ± 7.5 ^g^		20.1 ± 13.1 ^i^		11.8 ± 6.0	
Grade III (n = 3)	131.7 ± 9.0		41.5 ± 39.1		10.0 ± 10.4	
Lymphatic invasion						
Negative (n = 30)	59.2 ± 22	*p* < 0.0005	9.6 ± 10.9	NS	4.1 ± 6.2	NS
Positive (n = 9)	95.7 ± 27.9		20.2 ± 26.0		6.9 ± 7.6	
ER						
Positive (n = 24)	54.6 ± 20.9	*p* < 0.0005	6.7 ± 7.1	*p* < 0.01	1.5 ± 2.5	*p* < 0.0005
Negative (n = 15)	88.5 ± 25.3		20.6 ± 21.9		9.7 ± 7.8	
PR						
Positive (n = 12)	57.7 ± 23.0	NS	8.8 ± 8.3	NS	1.9 ± 2.8	NS
Negative (n = 27)	72.0 ± 29.0		13.5 ± 18.2		5.9 ± 7.4	
C-erBb-2						
Positive (n = 8)	67.6 ± 23.2	NS	13.9 ± 27.4	NS	4.7 ± 7.2	NS
Negative (n = 31)	67.6 ± 29.5		11.5 ± 11.1		4.7 ± 6.4	
Cancer-related death						
Alive (n = 30)	63.1 ± 25.9	*p* < 0.05	10.1 ± 11	NS	4.3 ± 6.3	NS
Death (n = 9)	92.2 ± 27.7		22.5 ± 31.5		6.8 ± 7.8	

CC = complex carcinoma; STC = simple tubular carcinoma; STPC = simple tubulopapillary carcinoma; SSC = simple solid carcinoma; AC = anaplastic carcinoma. (a) *p* < 0.0005 vs. SSC + AC; (b) *p* < 0.05 vs. SSC + AC; (c) *p* < 0.0005 vs. SSC + AC; (d) *p* < 0.0005 vs. SSC + AC; (e) *p* < 0.0005 vs. SSC + AC; (f) *p* < 0.0005 vs. grade III, (g) *p* < 0.0005 vs. grade III, (h) *p* < 0.0005 vs. grade III, (i) *p* < 0.0005 vs. grade III, (l) *p* < 0.05 vs. grade III.

## Data Availability

Not applicable.

## Supplementary Materials

The following supporting information can be downloaded at: https://www.mdpi.com/article/10.3390/ani13030505/s1, Figure S1: Immunohistochemical analysis of estrogen receptor (ER) expression with an anti-ER monoclonal antibody in canine mammary carcinomas. (**A**) ER+ tubular mammary carcinoma. Most neoplastic cells showed nuclear immunostaining. IHC, hematoxylin counterstain; scale bar 50 µm. (**B**) ER- tubular mammary carcinoma. Few neoplastic cell nuclei are positive for receptor expression. IHC, hematoxylin counterstain; scale bar 100 µm. Figure S2: Immunohistochemical analysis of progesterone receptor (PR) expression with anti-PR monoclonal antibody in canine mammary carcinomas. (**A**) PR+ tubular mammary carcinoma. Several neoplastic cells showed nuclear. IHC, hematoxylin counterstain; scale bar 100 µm. (**B**) PR- tubular mammary carcinoma. Few neoplastic cell nuclei are positive for receptor expression. IHC, hematoxylin counterstain; scale bar 100 µm. Figure S3. Immunohistochemical analysis of C-erbB-2 expression using a polyclonal antibody anti-C-erbB-2 in canine mammary carcinomas. (**A**) Tubular mammary carcinoma scored 1+. Faint incomplete membranous reactivity. IHC, hematoxylin counterstain; scale bar 50 µm. (**B**) Tubular mammary carcinoma scored 2+. Weak to moderate incomplete membranous reactivity. IHC, hematoxylin counterstain; scale bar 50 µm. (**C**) Tubular mammary carcinoma scored 3+ (C-erbB-2 overexpression). Strong and complete membranous reactivity. IHC, hematoxylin counterstain; scale bar 50 µm.

## References

[1] LeBleu V. (2015). Imaging the tumor microenvironment. Cancer J..

[2] Arneth B. (2019). Tumor Microenvironment. Medicina.

[3] Korneev K.V., Atretkhany K.N., Drutskaya M.S., Grivennikov S.I., Kuprash D.V., Nedospasov S.A. (2017). TLR-signaling and proinflammatory cytokines as drivers of tumorigenesis. Cytokine.

[4] Hsieh C.S., Lee H.M., Lio C.W. (2012). Selection of regulatory T cells in the thymus. Nat. Rev. Immunol..

[5] Whiteside T.L. (2018). FOXP3+ Treg as a therapeutic target for promoting anti-tumor immunity. Expert Opin. Ther. Targets.

[6] Bates G.J., Fox S.B., Han C., Leek R.D., Garcia J.F., Harris A.L., Banham A.H. (2006). Quantification of regulatory T cells enables the identification of high-risk breast cancer patients and those at risk of late relapse. J. Clin. Oncol..

[7] Gao Y., Tang J., Chen W., Li Q., Nie J., Lin F., Wu Q., Chen Z., Gao Z., Fan H. (2015). Inflammation negatively regulates FOXP3 and regulatory T-cell function via DBC1. Proc. Natl. Acad. Sci. USA.

[8] Zhu S., Lin J., Qiao G., Xu Y., Zou H. (2015). Differential regulation and function of tumor-infiltrating T cells in different stages of breast cancer patients. Tumour. Biol..

[9] Zhang C., Xu Y., Hao Q., Wang S., Li H., Li J., Gao Y., Li M., Li W., Xue X. (2015). FOXP3 suppresses breast cancer metastasis through downregulation of CD44. Int. J. Cancer.

[10] Kim J.H., Hur J.H., Lee S.M., Im K.S., Kim N.H., Sur J.H. (2012). Correlation of Foxp3 positive regulatory T cells with prognostic factors in canine mammary carcinomas. Vet. J..

[11] Carvalho M.I., Pires I., Prada J., Gregório H., Lobo L., Queiroga F.L. (2016). Intratumoral FoxP3 expression is associated with angiogenesis and prognosis in malignant canine mammary tumors. Vet. Immunol. Immunopathol..

[12] Sakai K., Maeda S., Yamada Y., Chambers J.K., Uchida K., Nakayama H., Yonezawa T., Matsuki N. (2018). Association of tumour-infiltrating regulatory T cells with adverse outcomes in dogs with malignant tumours. Vet. Comp. Oncol..

[13] Fontenot J.D., Rasmussen J.P., Williams L.M., Dooley J.L., Farr A.G., Rudensky A.Y. (2005). Regulatory T cell lineage specification by the forkhead transcription factor foxp3. Immunity.

[14] Sorenmo K.U., Rasotto R., Zappulli V., Goldschmidt M.H. (2011). Development, anatomy, histology, lymphatic drainage, clinical features, and cell differentiation markers of canine mammary gland neoplasms. Vet. Pathol..

[15] Goldschmidt M., Penã L., Rasotto R., Zappulli V. (2011). Classification and grading of canine mammary tumors. Vet. Pathol..

[16] Peña L., De Andres P.J., Clemente M., Cuesta P., Perez-Alenza M.D. (2013). Prognostic value of histological grading in noninflammatory canine mammary carcinomas in a prospective study with two-year follow-up: Relationship with clinical and histological characteristics. Vet. Pathol..

[17] Mahmoud S.M., Paish E.C., Powe D.G., Macmillan R.D., Lee A.H., Ellis I.O., Green A.R. (2011). An evaluation of the clinical significance of FOXP3(+) infiltrating cells in human breast cancer. Breast Cancer Res. Treat..

[18] Beha G., Brunetti B., Asproni P., Muscatello L.V., Millanta F., Poli A., Sarli G., Benazzi C. (2012). Molecular portrait-based correlation between primary canine mammary tumor and its lymph node metastasis: Possible prognostic-predictive models and/or stronghold for specific treatments?. BMC Vet. Res..

[19] Millanta F., Calandrella M., Bari G., Niccolini M., Vannozzi I., Poli A. (2005). Comparison of steroid receptor expression in normal, dysplastic, and neoplastic canine and feline mammary tissues. Res. Vet. Sci..

[20] Balkwill F.R., Capasso M., Hagemann T. (2012). The tumor microenvironment at a glance. J. Cell Sci..

[21] Hanahan D., Coussens L. (2012). Accessories to the crime: Functions of cells recruited to the tumor microenvironment. Cancer Cell.

[22] Fozza C., Longinotti M. (2011). T-cell traffic jam in Hodgkin’s lymphoma: Pathogenetic and therapeutic implications. Adv. Hematol..

[23] Guillerey C., Huntington N., Smyth M. (2016). Targeting natural killer cells in cancer immunotherapy. Nat. Immunol..

[24] De Oliveira L.O., De Oliveira R.T., Loretti A.P., Rodrigues R., Driemeier D. (2003). Aspectos epidemiológicos da neoplasia mamária canina. Acta. Sci. Vet..

[25] Sorenmo K. (2003). Canine mammary gland tumors. Vet. Clin. N. Am. Small Anim. Pract..

[26] Monteiro L.N., Dos Reis D.C., Salgado B.S., Cassali G.D. (2021). Clinical significance and prognostic role of tumor-associated macrophages infiltration according to histologic location in canine mammary carcinomas. Res. Vet. Sci..

[27] Parisi F., Tesi M., Millanta F., Gnocchi M., Poli A. (2021). M1 and M2 tumour-associated macrophages subsets in canine malignant mammary tumours: An immunohistochemical study. Res. Vet. Sci..

[28] De Souza T.A., de Campos C.B., De Biasi Bassani Gonçalves A., Nunes F.C., Monteiro L.N., de Oliveira Vasconcelos R., Cassali G.D. (2018). Relationship between the inflammatory tumor microenvironment and different histologic types of canine mammary tumors. Res. Vet. Sci..

[29] Estrela-Lima A., Araújo M.S., Costa-Neto J.M., Teixeira-Carvalho A., Barrouin-Melo S.M., Cardoso S.V., Martins-Filho O.A., Serakides R., Cassali G.D. (2010). Immunophenotypic features of tumor infiltrating lymphocytes from mammary carcinomas in female dogs associated with prognostic factors and survival rates. BMC Cancer.

[30] Kim J.H., Chon S.K., Im K.S., Kim N.H., Sur J.H. (2013). Correlation of tumor-infiltrating lymphocytes to histopathological features and molecular phenotypes in canine mammary carcinoma: A morphologic and immunohistochemical morphometric study. Can. J. Vet. Res..

[31] Serr I., Kral M., Scherm M.G., Daniel C. (2021). Advances in Human Immune System Mouse Models for Personalized Treg-Based Immunotherapies. Front. Immunol..

[32] Franzoni M.S., Brandi A., de Oliveira Matos Prado J.K., Elias F., Dalmolin F., de Faria Lainetti P., Prado M.C.M., Leis-Filho A.F., Fonseca-Alves C.E. (2019). Tumor-infiltrating CD4+ and CD8+ lymphocytes and macrophages are associated with prognostic factors in triple-negative canine mammary complex type carcinoma. Res. Vet. Sci..

[33] Pinard C.J., Lagree A., Lu F.I., Klein J., Oblak M.L., Salgado R., Cardenas J.C.P., Brunetti B., Muscatello L.V., International Immuno-Oncology Biomarker Working Group (2022). Comparative Evaluation of Tumor-Infiltrating Lymphocytes in Companion Animals: Immuno-Oncology as a Relevant Translational Model for Cancer Therapy. Cancers.

[34] Muscatello L.V., Avallone G., Brunetti B., Bacci B., Foschini M.P., Sarli G. (2022). Standardized approach for evaluating tumor infiltrating lymphocytes in canine mammary carcinoma: Spatial distribution and score as relevant features of tumor malignancy. Vet. J..

[35] Elkord E., Alcantar-Orozco E.M., Dovedi S.J., Tran D.Q., Hawkins R.E., Gilham D.E. (2010). T regulatory cells in cancer: Recent advances and therapeutic potential. Expert Opin. Biol. Ther..

[36] Nishikawa H., Sakaguchi S. (2014). Regulatory T cells in cancer immunotherapy. Curr. Opin. Immunol..

[37] Whiteside T.L. (2014). Induced regulatory T cells in inhibitory microenvironments created by cancer. Expert Opin. Biol. Ther..

[38] Merlo A., Casalini P., Carcangiu M.L., Malventano C., Triulzi T., Menard S., Tagliabue E., Balsari A. (2009). FOXP3 expression and overall survival in breast cancer. J. Clin. Oncol..

[39] Yamaguchi T., Sakaguchi S. (2006). Regulatory T cells in immune surveillance and treatment of cancer. Semin Cancer Biol..

[40] Zou W. (2006). Regulatory T cells, tumour immunity and immunotherapy. Nat. Rev. Immunol..

[41] Oh S.Y., Ryu H.H., Yoo D.Y., Hwang I.K., Kweon O.K., Kim W.H. (2014). Evaluation of FOXP3 expression in canine mammary gland tumours. Vet. Comp. Oncol..

[42] Tarighati E., Keivan H., Mahani H. (2022). A review of prognostic and predictive biomarkers in breast cancer. Clin. Exp. Med..

[43] Bertozzi S., Londero A.P., Seriau L., Di Vora R., Cedolini C., Mariuzzi L., Begum G. (2018). Biomarkers in Breast Cancer. Bio-Marker Indicator of Abnormal Physiological Process.

[44] Queiroga F.L., Raposo T., Carvalho M.I., Prada J., Pires I. (2011). Canine mammary tumours as a model to study human breast cancer: Most recent findings. In Vivo.

[45] Nguyen F., Peña L., Ibisch C., Loussouarn D., Gama A., Rieder N., Belousov A., Campone M., Abadie J. (2018). Canine invasive mammary carcinomas as models of human breast cancer. Part 1: Natural history and prognostic factors. Breast Cancer Res. Treat..

[46] Raposo T.P., Arias-Pulido H., Chaher N., Fiering S.N., Argyle D.J., Prada J., Pires I., Queiroga F.L. (2017). Comparative aspects of canine and human inflammatory breast cancer. Semin. Oncol..

[47] Peña L., Gama A., Goldschmidt M.H., Abadie J., Benazzi C., Castagnaro M., Díez L., Gärtner F., Hellmén E., Kiupel M. (2014). Canine mammary tumors: A review and consensus of standard guidelines on epithelial and myoepithelial phenotype markers, HER2, and hormone receptor assessment using immunohistochemistry. Vet. Path..

[48] Lamb C.A., Vanzulli S.I., Lanari C. (2019). Hormone receptors in breast cancer: More than estrogen receptors. Receptores hormonales en cáncer de mama: Receptores de estrógenos y algo más. Medicina.

[49] Payne S.J., Bowen R.L., Jones J.L., Wells C.A. (2008). Predictive markers in breast cancer--the present. Histopathology.

[50] Cao W., Zhang B., Liu Y., Li H., Zhang S., Fu L., Niu Y., Ning L., Cao X., Liu Z. (2007). High-level SLP-2 expression and HER-2/neu protein expression are associated with decreased breast cancer patient survival. Am. J. Clin. Pathol..

[51] Reix N., Malina C., Chenard M.P., Bellocq J.P., Delpous S., Molière S., Sevrin A., Neuberger K., Tomasetto C., Mathelin C. (2016). A prospective study to assess the clinical utility of serum HER2 extracellular domain in breast cancer with HER2 overexpression. Breast Cancer Res. Treat..

