# Supplementary material for: Confirmation of the Prognostic Value of Foxp3+ Cells in Canine Mammary Tumors
Source: Animals (Basel). 2023 Jan 31;13(3):505. doi: 10.3390/ani13030505 (PMC9913641; doi:10.3390/ani13030505)
Supplement: Supplementary file 1 [file animals-13-00505-s001.zip › animals-2142247-supplementary.pdf]

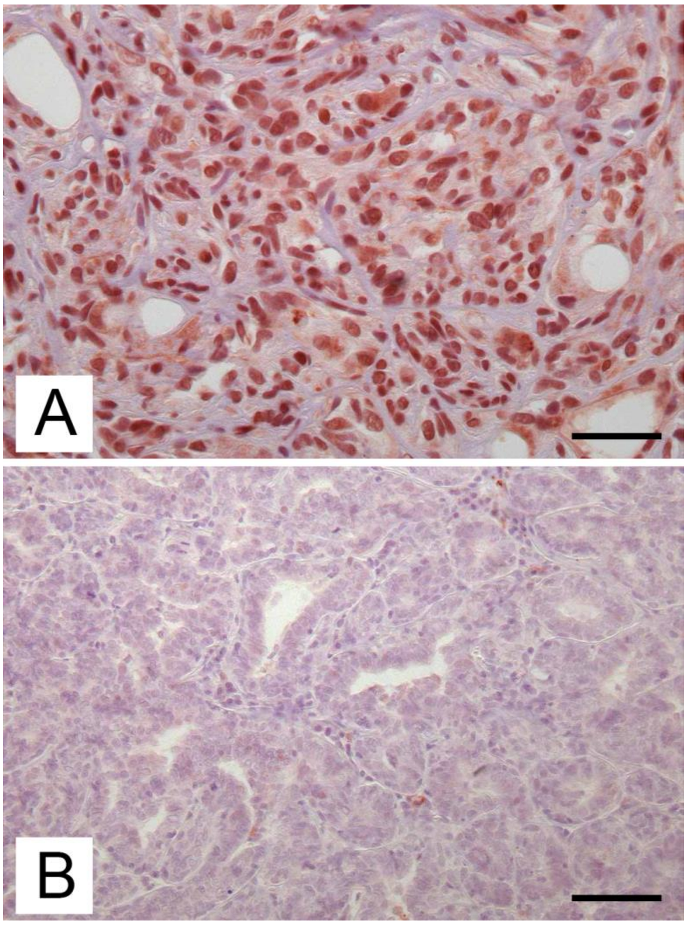

Figure S1. Immunohistochemical analysis of estrogen receptor (ER) expression with an anti-ER monoclonal antibody in canine mammary carcinomas. A) ER+ tubular mammary carcinoma. Most neoplastic cells showed nuclear immunostaining. IHC, hematoxylin counterstain; scale bar 50 μm. B) ER- tubular mammary carcinoma. Few neoplastic cell nuclei are positive for receptor expression. IHC, hematoxylin counterstain; scale bar 100 μm.

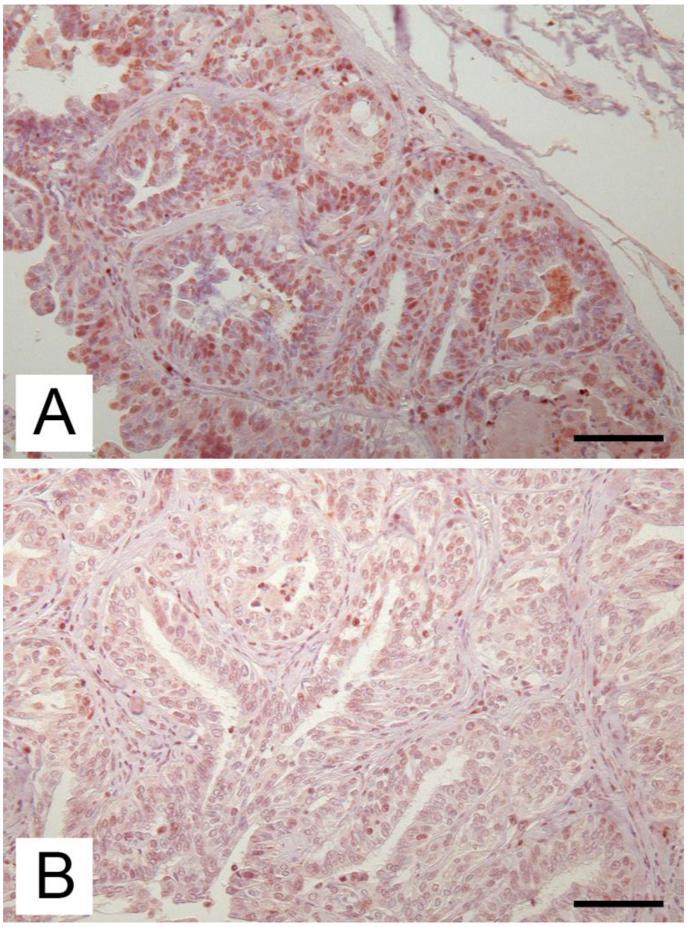

Figure S2. Immunohistochemical analysis of progesterone receptor (PR) expression with anti-PR monoclonal antibody in canine mammary carcinomas. A) PR+ tubular mammary carcinoma. Several neoplastic cells showed nuclear IHC, hematoxylin counterstain; scale bar 100 µm. B) PR- tubular mammary carcinoma. Few neoplastic cell nuclei are positive for receptor expression. IHC, hematoxylin counterstain; scale bar 100 µm.

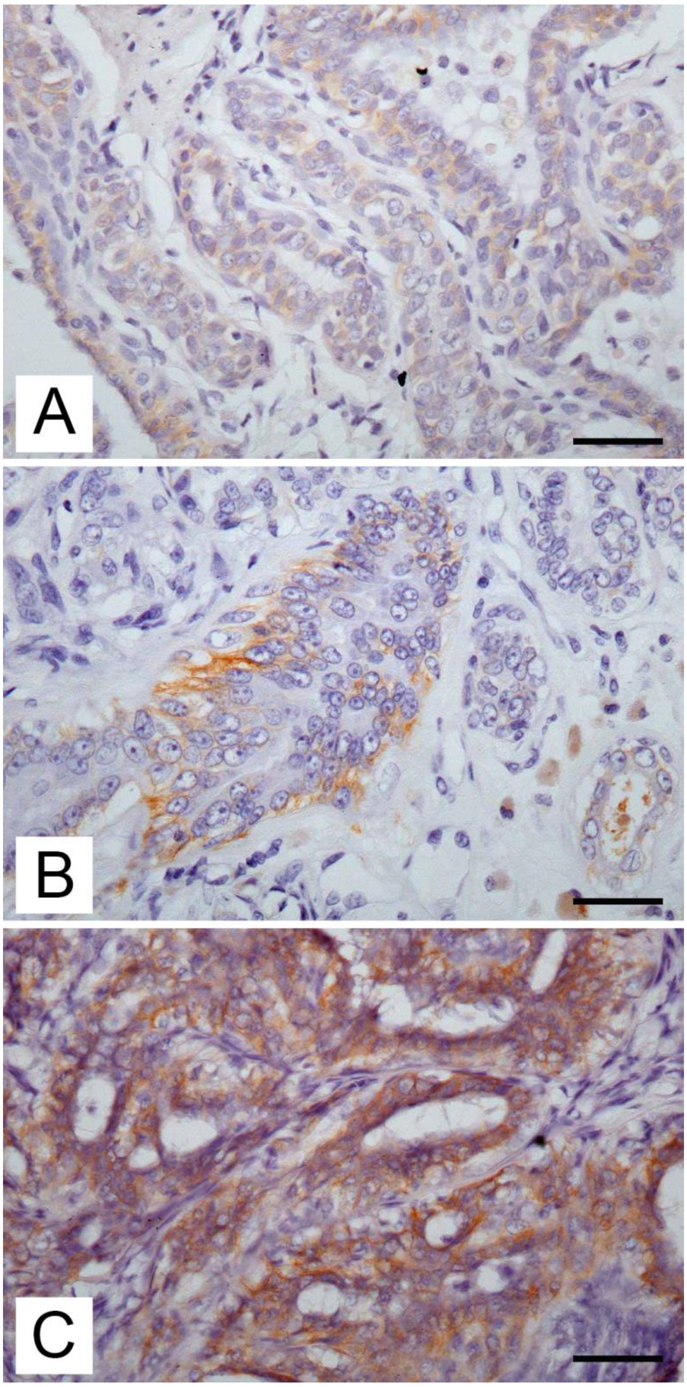

Figure S3. Immunohistochemical analysis of C-erbB-2 expression using a polyclonal antibody anti-C-erbB-2 in canine mammary carcinomas. A) Tubular mammary carcinoma scored 1+. Faint incomplete membranous reactivity. IHC, hematoxylin counterstain; scale bar 50 μm. B) Tubular mammary carcinoma scored 2+. Weak to moderate incomplete membranous reactivity. IHC, hematoxylin counterstain; scale bar 50 μm. C) Tubular mammary carcinoma scored 3+ (C-erbB-2 overexpression). Strong and complete membranous reactivity. IHC, hematoxylin counterstain; scale bar 50 μm.
